# Supplementary figures and images for: Assessment of emissions and potential occupational exposure to carbon monoxide during biowaste composting
Source: PLoS One. 2024 Mar 8;19(3):e0290206. doi: 10.1371/journal.pone.0290206 (PMC10923444; doi:10.1371/journal.pone.0290206)

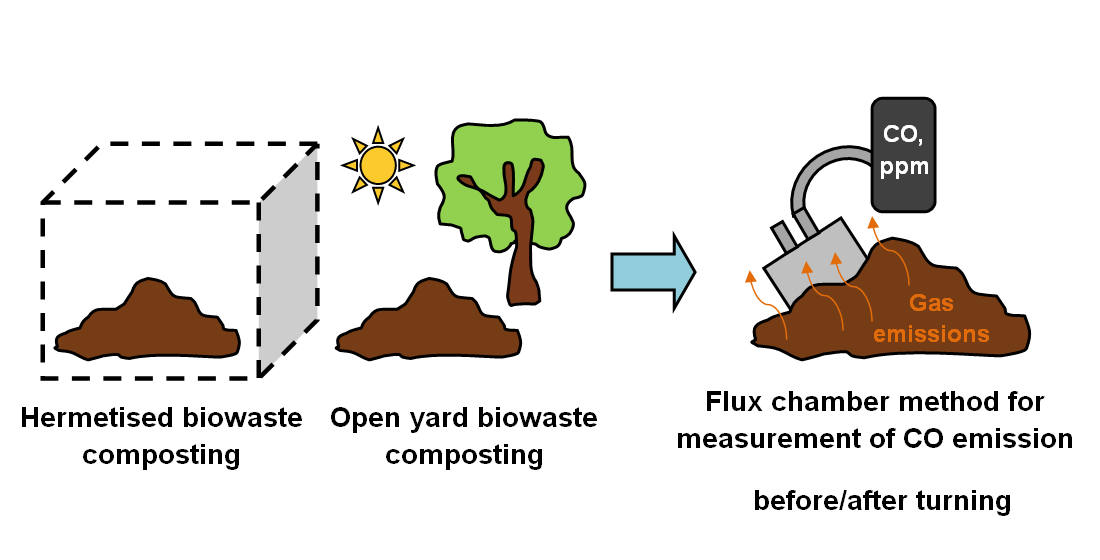

Supplement: S1 Graphical abstract — (TIF) [file pone.0290206.s002.tif]
